# The spatial spread of schistosomiasis: A multidimensional network model applied to Saint-Louis region, Senegal

**DOI:** 10.1016/j.advwatres.2016.10.012

**Published:** 2017-10

**Authors:** Manuela Ciddio, Lorenzo Mari, Susanne H. Sokolow, Giulio A. De Leo, Renato Casagrandi, Marino Gatto

**Affiliations:** a Dipartimento di Elettronica, Informazione e Bioingegneria, Politecnico di Milano, 20133 Milano, Italy; b Hopkins Marine Station, Stanford University, Pacific Grove, CA 93950, United States; c Marine Science Institute, University of California, Santa Barbara, CA 93106, United States

**Keywords:** Neglected tropical diseases, Multidimensional network model, Mobile phone records, Spatial connectivity, Metapopulations

## Abstract

Schistosomiasis is a parasitic, water-related disease that is prevalent in tropical and subtropical areas of the world, causing severe and chronic consequences especially among children. Here we study the spatial spread of this disease within a network of connected villages in the endemic region of the Lower Basin of the Senegal River, in Senegal. The analysis is performed by means of a spatially explicit metapopulation model that couples local-scale eco-epidemiological dynamics with spatial mechanisms related to human mobility (estimated from anonymized mobile phone records), snail dispersal and hydrological transport of schistosome larvae along the main water bodies of the region. Results show that the model produces epidemiological patterns consistent with field observations, and point out the key role of spatial connectivity on the spread of the disease. These findings underline the importance of considering different transport pathways in order to elaborate disease control strategies that can be effective within a network of connected populations.

## Highlights

• Environmental and social connectivity has a key role on the spread of schistosomiasis.
• A coupled human-snail-larval system is applied in a connected environment.
• Water contact patterns are estimated by coupling CDRs with hydrological information.
• The implementation of a comprehensive approach is important for fighting the disease.

## 1 Introduction

Schistosomiasis is an acute and chronic water-related disease caused by parasitic worms that affects about 250 million individuals worldwide (WHO Expert Committee, 2002). As one of the commonest and most devastating parasitic diseases, it is second only to malaria, inducing severe consequences to 20 million people (Kheir et al., 1999) and being directly responsible for 12,000 deaths yearly (Lozano et al., 2012). With an estimated burden of 4.5 million disability-adjusted life years (Fenwick, 2012, WHO Expert Committee, 2002), schistosomiasis is prevalent in tropical and subtropical areas, especially in poor communities without access to safe drinking water and adequate sanitation. It is estimated that at least 90% of those requiring treatment for schistosomiasis live in Africa (WHO Expert Committee, 2002).

Water has a key role in schistosomiasis transmission and spread. Human-to-environment transmission occurs when infected people contaminate freshwater bodies with their excreta containing parasite eggs. Environment-to-human transmission occurs when people are exposed to infested water during routine activities, ranging from agricultural to domestic and from occupational to recreational. Therefore, the disease is especially prevalent in rural communities. Lack of hygiene and certain play habits make school-aged children particularly vulnerable to infection, an aspect which must be regarded with care, because schistosomiasis may induce severe health consequences in absence of adequate treatments.

**Fig. 1:**
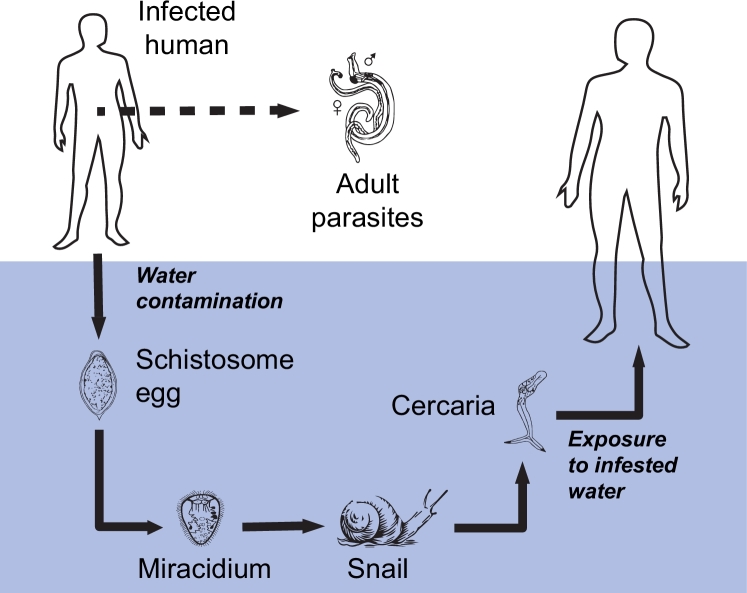
Schistosoma life cycle. Adult schistosomes within infected human hosts produce eggs, which are shed in the environment through excreta. The eggs that reach freshwater can hatch into miracidia and infect species-specific intermediate snail hosts. Infective snails shed free-swimming cercariae that can penetrate human skin and eventually develop into reproductive worms. See text for further details about transmission.

There are two major forms of schistosomiasis – intestinal and urogenital – caused by six species of blood flukes belonging to the genus *Schistosoma*, of which *S. haematobium, S. mansoni* and *S. japonicum* are the three most important ones (Colley et al., 2014). People become infected when larval forms of the parasite penetrate their skin during contact with infested water. The freely swimming, short-lived larval stages of the parasites are known as cercariae and are shed by some species of freshwater snails belonging to the genus *Bulinus* (for *S. haematobium*), *Biomphalaria* (for *S. mansoni*) or *Oncomelania* (for *S. japonicum*), which serve as species-specific obligate intermediate hosts for the parasites. Within the human body, the larvae need 5–7 weeks to develop into sexually mature adult schistosomes (Colley et al., 2014). Adult worms can live for a few years in the human blood vessels, where the females produce hundreds to thousands of fertilized eggs daily. Some of the eggs become trapped in body tissues, causing immune reactions and progressive damage to internal organs (e.g. liver), other leave the human host by being shed in the environment through the feces (*S. mansoni* or *S. japonicum*) or urine (*S. haematobium*) to continue the parasite’s life-cycle. The eggs released out of the human body that reach freshwater can hatch into a second short-lived larval form of the parasite, the miracidia, that are infectious for snails. In the snail, miracidia undergo asexual replication for 4–6 weeks (the so-called prepatent period; Colley et al., 2014), then the snail becomes infective and starts shedding hundreds of cercariae per day into water. A sketched scheme of the parasite life cycle is shown in Fig. 1.

The analysis of the coupled dynamics of human, parasite and snail populations, together with the free-living stages involved in the parasite’s life cycle, are fundamental to describe and understand the transmission mechanisms of schistosomiasis. Previous studies have already shown that disease dynamics not only depend upon interactions between infectious agents and the hosts, but also that they are strongly affected by environmental factors (Gurarie and Seto, 2009; Perez-Saez et al., 2015). In addition, large-scale dynamics are better described by metapopulation models, which proved to be a powerful tool in order to understand disease persistence and infection intensity in human societies (Grenfell, Harwood, 1997, Hagenaars, Donnelly, Ferguson, 2004). In the case of schistosomiasis, the movement of infectious agents can occur via various transport processes involving hosts and pathogens, including human mobility, larval transport along canals and streams, and snails dispersal through hydrological interconnections. The spread of the disease under study is thus the result of the interplay between various mechanisms acting at different spatial and temporal scales. On the human host side, social connections provide a pathway for adult parasite transport while people travel between endemic and non-endemic areas. This movement can involve very large spatial scales in ways that are often difficult to predict (Remais, 2010), and constitutes an effective transmission mechanism provided that disease-transmitting snails live in the visited areas. On the snail and parasite side, connectivity via physical processes (hydrological transport and animal dispersal) increases the risk of larval and snail propagation over shorter spatial scales. As an example, all over the world, an estimated 63 million people at risk for schistosomiasis live in irrigated environments, with an increased relative risk of urinary and intestinal schistosomiasis of 1.1 and 4.7, respectively, compared with non-irrigated environments (Steinmann et al., 2006).

Here we explore a spatially realistic metapopulation model (Section 2), in which schistosomiasis spreads within a network of connected villages. The model is applied to the area of the Lower Basin of the Senegal River, in the northern part of Senegal (for more details, see Section 3). Social and environmental interconnections link villages through human mobility (direct transport of parasites by humans) and hydrology (a pathway for larvae and snails). Results are presented in Section 4, while a set of concluding remarks closes the paper (Section 5).

## 2 The model

The basis of our analysis is a spatially explicit nonlinear model that accounts for local epidemiological dynamics, human mobility, snail dispersal and hydrological transport of schistosome larvae. At the local scale, the model extends the work presented in Ciddio et al. (2015) by including the dynamics of the larval stages of parasites. The system of differential equations is expressed in terms of the human population size (*N_v_*) and the total number of parasites (*P_v_*) within human hosts living in each village (subscript *v*), the density of susceptible, exposed, and infectious snails (*S_w_, E_w_, I_w_*) in the freshwater point (subscript *w*), and the concentration of cercariae (*C_w_*) and miracidia (*M_w_*) in the freshwater body. Early studies on the heterogeneity of schistosomiasis transmission (Barbour, 1978) already introduced a partitioning between human and animal host populations, but did not consider physical connectivity through the environment, an approach followed also in later works (Gurarie, King, 2005, Gurarie, King, Wang, 2010, Woolhouse, Etard, Dietz, Ndhlovu, Chandiwana, 1998, Woolhouse, Watts, Chandiwana, 1991). On the other hand, other studies (Gurarie and Seto, 2009; Perez-Saez et al., 2015; Xu et al., 2006) did consider the role of environmental connectivity (typically through larval dispersal alone), while at the same time neglecting the possible spatial mismatch between villages and water contact points (but see Remais, 2010, in which, however, snail dispersal is neglected). In our work, instead, *n_v_* villages and *n_w_* water points constitute two distinct sets of nodes of a fully coupled, multi-layered (multidimensional, sensu Boccaletti et al., 2014), spatially explicit network. Epidemiological dynamics can be described by the following set of $\left(2n_{v}+5n_{w}\right)$ ordinary differential equations:

(1) $$\left\{ \begin{array}{c} {\dot{N}}_{v}=\mu_{H}\left(H_{v}-N_{v}\right)-\alpha P_{v}\\ {\dot{P}}_{v}=F_{v}N_{v}-\left(\mu_{H}+\mu_{P}+\alpha \right)P_{v}-\alpha \frac{k+1}{k}\frac{P_{v}^{2}}{N_{v}}\\ {\dot{S}}_{w}=\nu S_{w}\left[1-\gamma_{w}\left(S_{w}+E_{w}+I_{w}\right)\right]-\mu_{S}S_{w}-\rho M_{w}S_{w}+D_{w}^{S}\\ {\dot{E}}_{w}=\rho M_{w}S_{w}-\left(\mu_{S}+\eta \right)E_{w}-\delta E_{w}+D_{w}^{E}\\ {\dot{I}}_{w}=\delta E_{w}-\left(\mu_{S}+\eta \right)I_{w}+D_{w}^{I}\\ {\dot{C}}_{w}=\zeta I_{w}-\mu_{C}C_{w}+L_{w}^{C}\\ {\dot{M}}_{w}=G_{w}-\mu_{M}M_{w}+L_{w}^{M}. \end{array}\right.$$

At each village *v*, human hosts are characterized by a constant recruitment *μ_H_H_v_* (with *H_v_* being the community size and *μ_H_* being the per capita natality rate), and two loss contributions due to non-schistosomiasis-related deaths (with rate *μ_H_*) and mortality induced by parasites (with *α* being a constant determining the pathogenicity of the parasite to the human host; Anderson and May, 1978). The human-to-schistosome interaction is modeled as a macroparasitic infection (Anderson and May, 1992), assuming that parasites are unevenly distributed among human hosts according to a negative binomial distribution with clumping parameter *k* (Feng et al., 2002). Parasite acquisition by human hosts is determined by contact with cercariae at infested water points, as described by the force of infection $F_{v},$ expressed as

(2) $$F_{v}=\beta \sum_{j=1}^{n_{w}}\Omega_{vw}C_{w}$$

where *β* is the human exposure rate, assumed to be uniform in space for the sake of simplicity, and $\Omega =[\Omega_{vw}]$ is a contact matrix describing the probability that an inhabitant of village *v* enters in contact with water point *w* (details in Section 3). The total number of adult parasites *P_v_* is limited by the natural host mortality (*μ_H_*), because every human dying will kill the hosted parasites, the per capita parasite mortality within the host (*μ_P_*), and the disease-induced mortality of humans ($\alpha +\alpha \left(k+1\right)/k\cdot P_{v}/N_{v}$).

Disease dynamics in the snail host are described via a compartmental SEI-like model, accounting for density-dependent mechanisms related to snails dynamics (Feng, Li, Milner, 2002, Mangal, Paterson, Fenton, 2010). In particular, it is assumed that infected snails are unable to reproduce and that snails are born (uninfected) according to a logistic recruitment function, with *ν* being the intrinsic natality rate and *γ_w_* capturing the site-specific effect of density dependence. Susceptible snails die at rate *μ_S_*. Recruitment of snails into the exposed compartment is assumed to be proportional to miracidial concentration through parameter *ρ*, which represents the exposure rate of susceptible snails. The model introduces a delay 1/*δ* between infection (i.e. transition from the susceptible to the exposed compartment) and onset of infectiousness (i.e. transition from the exposed to the infectious compartment), when infectious snails start shedding cercariae into the freshwater. In addition to a natural death rate *μ_S_*, infected snails (both exposed and infectious) are also subject to a disease-induced death rate *η*. Spatial dynamics are also considered, assuming that snails can move along the hydrological network according to the following functions:

(3) $$\begin{array}{c} D_{w}^{S}=\sum_{i=1}^{n_{w}}m_{i}^{S}Z_{iw}\frac{V_{i}}{V_{w}}S_{i}-m_{w}^{S}S_{w},\\ D_{w}^{E}=\sum_{i=1}^{n_{w}}m_{i}^{E}Z_{iw}\frac{V_{i}}{V_{w}}E_{i}-m_{w}^{E}E_{w},\\ D_{w}^{I}=\sum_{i=1}^{n_{w}}m_{i}^{I}Z_{iw}\frac{V_{i}}{V_{w}}I_{i}-m_{w}^{I}I_{w}, \end{array}$$

where *V_w_* is the freshwater volume of water point *w*, $m_{w}^{S,E,I}$ are the possibly site-specific and infection-specific snail movement rates, and *Z_iw_* is the element of the snail dispersal matrix obtained by transposing the connectivity matrix $Z=[Z_{wi}],$ a row-stochastic matrix (i.e. $\sum_{i=1}^{n_{w}}Z_{wi}=1\;\forall i$) that describes the probability that a snail moves between any two water points *w* and *i*.

As for the free-living larval stages of the schistosome, their concentrations in the freshwater environment is dynamically determined by the balance between mortality, shedding, and hydrological transport. In particular, cercariae are assumed to die at spatially uniform rate *μ_C_* and are shed by infective snails at rate *ζ*, whereas miracidia die at rate *μ_M_* and mature from the eggs shed by infected humans, which are proportional to the total number of adult parasite pairs hosted therein, according to the following function:

(4) $$G_{w}=\frac{\chi }{2V_{w}}\sum_{v=1}^{n_{v}}P_{v}\Omega_{vw},$$

where *χ* is the human contamination rate (as specified in Section 3). Spatial interactions due to hydrological connectivity are formulated as:

(5) $$\begin{array}{c} L_{w}^{C}=\sum_{i=1}^{n_{w}}l_{i}^{C}T_{iw}\frac{V_{i}}{V_{w}}C_{i}-l_{w}^{C}C_{w},\\ L_{w}^{M}=\sum_{i=1}^{n_{w}}l_{i}^{M}T_{iw}\frac{V_{i}}{V_{w}}M_{i}-l_{w}^{M}M_{w}, \end{array}$$

where $l_{w}^{C,M}$ are the possibly site-specific larval transport rates, and *T_iw_* is the element of the larval transport matrix obtained by transposing the connectivity matrix $T=[T_{wi}],$ another row-stochastic matrix describing the probability that a cercaria/miracidium is transported from water point *w* to water point *i*. Note that we do not consider explicitly increased larval mortality during transport, which has instead been accounted for in other approaches (Gurarie and Seto, 2009). Given the regular geometry of our hydrological network (details in Section 3) and the assumed movement mechanisms, in our modelling framework this contribution is the same at every water node *w*, since miracidia/cercariae are passively transported from each *w* to its downstream neighbor. Larval survival is then equally accounted for in the mortality rates *μ_C_* and *μ_M_*, defined at the daily time scale.

## 3 Application of the model to Saint-Louis region

The focal area of this study is the Lower Basin of the Senegal River, in Senegal. This area is particularly interesting because of the presence of the Diama Dam, which altered the environment and increased the risk of infection since it was built in the 1980s (Sow et al., 2002). It was designed to control the Senegal River regime and to ensure permanent water availability: in fact, the dam blocks the intrusion of salt water from the ocean, making the impounded river a stable reservoir of freshwater for people living in the region of Saint-Louis. At the same time, the dam created a suitable habitat for the snails hosting schistosomiasis and resulted in persistently high infection levels in the villages along the Senegal River and its tributaries (Talla et al., 1990). Moreover, the dam interfered with the life cycle of the prawn *Macrobrachium vollenhovenii*, an effective predator of the snails, whose key role as potential biological control agent has been the object of recent studies (Alkalay, Rosen, Sokolow, Faye, Faye, Aflalo, Jouanard, Zilberg, Huttinger, Sagi, 2014, Sokolow, Lafferty, Kuris, 2014, Sokolow, et al., 2015).

**Fig. 2:**
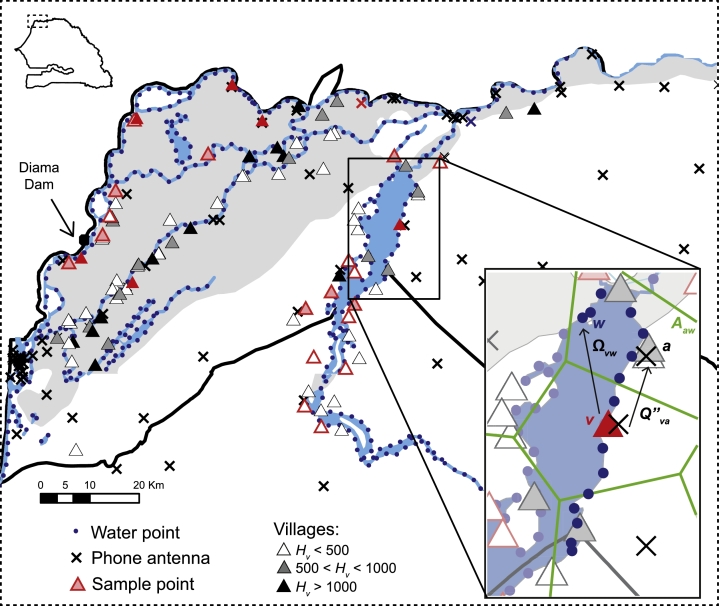
Our study area in the Lower Basin of the Senegal River. Within the study area, located in the northern part of Senegal, there are 90 villages (triangles) and 396 water points (blue points). Villages and water points are linked by contact patterns driven by human mobility, which is estimated from anonymized mobile phone traces left by mobile phone users logged in at antenna sites (crosses). Water points are also linked with each other through hydrological connectivity. Snail densities are estimated on the basis of a few sample points, where prevalence data of schistosomiasis are also available (red triangles). The grey-shaded territory indicates the fraction of land subject to inundation during floods. In the inset, a sketch of water contact patterns extraction limited to the area of Lac de Guiers is presented: the water contact matrix *Ω_vw_* is obtained from the mobility matrix $Q_{va}^{\prime \prime }$ describing village-to-antenna movement and the adjacency matrix *A_aw_* describing the proximity between water points and antennas, according to their influence area (green polygon). See details in the text. (For interpretation of the references to colour in this figure legend, the reader is referred to the web version of this article.)

We use the model presented in Section 2 to simulate the dynamics of schistosomiasis transmission in a multidimensional network system (Boccaletti et al., 2014) properly tailored to the study area, represented in Fig. 2. The network includes two different sets of nodes: the first set consists of $n_{v}=90$ villages located along the main water bodies of the region, selected by the Upstream Alliance (http://www.theupstreamalliance.org/) for the collection of demographic, prevalence and snail counts data; the second, of $n_{w}=396$ water points obtained through a discretization of the underlying hydrological network (data available from DIVA-GIS, http://www.diva-gis.org/gdata); specifically, we defined water points through an arbitrary (uniform) spatial discretization of canals, streams and lake perimeters (every ≈ 2 km). These two sets of nodes are linked by two different sets of edges, representing layers of spatial connectivity: the first layer describes the interactions between human communities and water points (human-to-water contact patterns); the second, of snail dispersal and larval transport along rivers and canals (water-to-water contact patterns, i.e. hydrological connectivity). A detailed description of model implementation and parametrization is reported below.

### 3.1 Human population

Population size of each village (*H_v_*) is available thanks to surveys conducted by the Upstream Alliance. In absence of sampled data, the number of resident people is obtained from a high-resolution population distribution map elaborated within the AfriPop project, which is part of the WorldPop project (data available online at http://www.worldpop.org.uk/). AfriPop data include 2014 estimates of population distribution with a spatial resolution of 30 arcsec (approx 100 m at the equator), and national totals adjusted to match United Nations estimates. As for the few villages excluded from the survey (19 villages), population size is computed by summing the 2014 population estimates of the grid squares that fall within a radius of 1 km from the geographical coordinates of the centroid of the village. To parameterize the demographic part of the model, we consider that the life expectancy of Senegalese people is 61 years (CIA, 2014), thus we set $\mu_{H}=4.5\cdot 10^{-5}$/day.

### 3.2 Snail population

Proxies of snail densities are also based on the results of relative abundance counts operated by the Upstream Alliance in a subset of water points (see again Fig. 2). We consider three qualitatively different water habitats for snails within the pilot area, i.e. lakes (H1), canals (H2) and main rivers (H3), and set the carrying capacity in absence of the parasite to the spatial average of the sampled values in each of the three habitats. According to the values obtained in the Upstream Alliance’s survey, we set 1 snail/m^3^ in every access point along the lakes (hence *γ_w_* = 1 snails${}^{-1}$ m^3^), 9 snails/m^3^ along the canals (hence *γ_w_* = 0.11 snails${}^{-1}$ m^3^), and 11 snails/m^3^ along the main Senegalese course of the Senegal river (hence *γ_w_* = 0.09 snails${}^{-1}$ m^3^). We also consider a fourth qualitative kind of habitat in the area of Saint-Louis, which is the chief town of the homonymous region. In this area, about twenty kilometers south of the dam, water is particularly salty because of the intrusion coming from the ocean, which is actually the reason why the dam was built to protect the upper part of the region, and this makes the habitat unsuitable for the snails. For the sake of simplicity, we thus assume that snails cannot live in the water points near the town, and set *S_w_, E_w_*, and *I_w_* to be always null within a radius of 20 km from Saint-Louis. As for the parameterization of the snail compartments in the model, the baseline mortality rates of snails can be evaluated as the inverse of their average lifespans: 1 year for uninfected snails (hence $\mu_{S}=2.7\cdot 10^{-3}$/day) and 2 months for infected snails (hence $\eta =1.37\cdot 10^{-2}$/day) (Feng et al., 2004). The intrinsic natality rate is strongly dependent on the environmental conditions (Webbe, 1962) and exposition to schistosomes (Mangal et al., 2010). Assuming that each snail produces at most 180 eggs in 10 weeks (Mangal et al., 2010), with a hatching rate of about 30%, we set ν=0.7/day. The exposure rate of susceptible snails is set to $\rho =5\cdot 10^{-5}$ m^3^/miracidium/day (Feng et al., 2004), while the average duration of the prepatent period (i.e. the delay between infection and onset of infectiousness) is assumed to be about 2 weeks (Feng, Eppert, Milner, Minchella, 2004, Gryseels, Polman, Clerinx, Kestens, 2006) (hence $\delta =6.7\cdot 10^{-2}$/day).

### 3.3 Adult parasites and larval stages

The life expectancy of adult parasites within human hosts is about 5 years (Feng, Eppert, Milner, Minchella, 2004, Gryseels, Polman, Clerinx, Kestens, 2006) (hence $\mu_{P}=5.5\cdot 10^{-4}$/day). According to previous studies conducted in other endemic regions of Africa and South America, parasite-induced mortality *α* is assumed to be 1.1$\cdot 10^{-7}$/day (Kheir et al., 1999), and the clumping parameter *k* of the negative binomial distribution of parasites within human hosts is estimated to be 0.243 (Feng et al., 2004). As for the larval stages of the schistosomes, the average number of cercariae daily released by one infected snail is 350 (Feng et al., 2004), thus ζ=350 cercariae/(snail ·  day). The larvae have very short lifespans: ≈ 26 hours for cercariae (hence $\mu_{C}=0.91$/day), and less than 8 hours for miracidia (hence $\mu_{M}=3.04$/day) (May and Anderson, 1979).

### 3.4 Human mobility and water contact patterns

The first layer of spatial connectivity is defined by human-to-water contact patterns, which link the two sets of nodes of our network through the *n_v_* × *n_w_* contact matrix, $\Omega =[\Omega_{vw}]$. In the absence of comprehensive surveys on water use in the study area, contacts between human villages and water points have been estimated from proxies of human mobility and a geospatial analysis including human settlements and water bodies.

Human mobility patterns, in particular, have been derived from anonymized, low-resolution movement routes of Sonatel mobile phone users collected for one year, from January 1 to December 31, 2013. Sonatel is the main telecommunications provider of Senegal, with more than 9 million subscribers in the whole country. A subset of the mobile phone dataset (with data for about 150,000 anonymous users) was released in the context of the Data for Development (D4D) challenge promoted by Orange and Sonatel in 2014 (http://www.d4d.orange.com/en/home). Selected research teams were subsequently granted access to the full dataset, containing more than 15 billion Call Detail Records (CDRs). Information associated with each CDR includes the anonymous identifier of the user placing the call, the location of the antenna from where the call was initiated and a timestamp. The estimation of the entries of the contact matrix **Ω** requires further steps to elaborate this information, which needs to be rescaled from the antenna level to our network structure.

As a first step towards obtaining **Ω**, we preliminary build a row-stochastic mobility matrix (**Q**) accounting for human movement between antennas (crosses in Fig. 2). The entries *Q_ij_* of this *n_A_* × *n_A_* matrix ($n_{A}=1666$ being the number of antenna towers in Senegal) can be defined as the fraction of time spent close to antenna *j* by people usually “living” close to antenna *i* (see below). We use the number of phone calls made by a user as a proxy for the time spent in a given location. With this assumption, the entries of **Ω** are simply proportional to the number of phone calls made by users usually resident nearby antenna *i* while being close to antenna *j*. CDRs are used to identify the ‘home’ antenna for each anonymous user: such antenna being defined as the antenna site where most calls made by a user occur during night hours (from 7 pm to 7 am) over the whole dataset. Following this procedure, it is possible to determine that in 2013 425,548 Sonatel subscribers lived in the study area. Afterwards, the number of calls made from antenna *j* by users whose home antenna has been identified with *i* is extracted from the dataset for all antenna *i*. This number, divided by the total number of calls made by users whose home antenna is *i* (independently of the location where the call originates from), represents an estimate of the entries of the antenna-to-antenna mobility matrix **Q** (Mari et al., 2014).

As a second step, we define another human mobility matrix (**Q′**) describing village-to-antenna movement. To do so, the three antennas closest to each village *v* (say *i, j* and *h*) are selected. The *v*th row of matrix **Q′** is then obtained as a convex combination of the *i*th, *j*th and *h*th rows of **Q**, with weights proportional to $D_{va}^{-2},$ where *D_va_* is the distance between village *v* and antenna *a* (*a* ∈ {*i, j, h*}). We note that **Q′** is a *n_v_* × *n_A_* matrix, i.e. it accounts also for outgoing mobility fluxes that are not confined within our focal region. A reduced *n_v_* × *n_a_* village-to-antenna mobility matrix **Q′′** is readily obtained from **Q′** by selecting only the columns that refer to the *n_a_* antennas located within the study area; the rows of **Q′′** can then be suitably normalized to ensure that they sum up to one. Neglecting mobility fluxes directed to/originating from other regions of Senegal might seem like an oversimplification. However, CDRs show that, on average, ≈87% of the human mobility fluxes originating from the antennas encompassed in the study area are confined within our focal region; conversely, the antennas located in the study area attract, on average, only ≈ 0.5% of the mobility fluxes originating from other regions of Senegal. We can thus conclude that, with respect to human mobility, our study area represents a relatively isolated system.

As a third step, we define a *n_a_* × *n_w_* adjacency matrix (**A**) describing the proximity of each water point to each of the antennas located in the focal region. Specifically, *A_aw_* is set to unity if *a* is the antenna tower that is closest to water point *w*, to zero otherwise. In other words, $A_{aw}=1$ if and only if *w* lies within the influence area of *a*, an information which can be obtained via Voronoi partitioning of the focal region by using antenna sites as seeds (Aurenhammer, 1991). The water-contact matrix **Ω** is finally obtained as the product of **Q′′** and **A** (see next section). A sketch of water contact patterns construction is shown in the inset of Fig. 2 limited to a small part of the study area close to the main lake (Lac de Guiers).

### 3.5 Infection and contamination risk

By using the mobility and water proximity matrices just defined, the force of infection for humans living in village *v* can be expressed as

(6) $$F_{v}=\beta_{0}\sum_{a}Q_{va}^{\prime \prime }R_{a}\;,$$

where *β*_0_ is a baseline exposure rate, $Q_{va}^{\prime \prime }$ accounts for mobility of people living in *v* toward antenna *a*, and *R_a_* is the infection risk associated with being close to antenna *a* and its nearby water points. We assume that *R_a_* is an increasing linear function of the concentrations of cercariae infesting each of the water points lying in the influence area of *a*, i.e.

(7) $$R_{a}=\beta_{1}\sum_{w}A_{aw}C_{w}\;,$$

with *β*_1_ representing the probability that exposure to cercariae eventually results in human infection. Defining the synthetic exposure rate $\beta =\beta_{0}\beta_{1},$ we finally get

(8) $$F_{v}=\beta_{0}\beta_{1}\sum_{a}Q_{va}^{\prime \prime }\sum_{w}A_{aw}C_{w}=\beta \sum_{w}\Omega_{vw}C_{w}\;,$$

where $\Omega =Q^{\prime \prime }A$. The previous expression corresponds to the definition of the force of infection anticipated above in Eq. (2).

By using the same matrices, water contamination resulting from people shedding eggs/miracidia at water point *w* can be expressed as

(9) $$G_{w}=\frac{\chi_{0}}{V_{w}}\sum_{a}A_{aw}R_{a}^{\prime }\;,$$

where *χ*_0_ is a baseline contamination rate and $R_{a}^{\prime }$ is the relative contamination contribution associated with people moving close to antenna *a*, therefore possibly contacting its nearby water points. We assume that $R_{a}^{\prime }$ is an increasing linear function of the number of adult parasite pairs within human population moving from any village *v*, i.e.

(10) $$R_{a}^{\prime }=\chi_{1}\sum_{v}\frac{P_{v}}{2}Q_{va}^{\prime \prime }\;,$$

with *χ*_1_ representing the probability that parasite eggs released into freshwater eventually result in miracidia maturation. By introducing the synthetic contamination rate $\chi =\chi_{0}\chi_{1},$ we finally get

(11) $$G_{w}=\frac{\chi_{0}\chi_{1}}{2V_{w}}\sum_{a}A_{aw}\sum_{v}P_{v}Q_{va}^{\prime \prime }=\frac{\chi }{2V_{w}}\sum_{v}P_{v}\Omega_{vw}\;,$$

which corresponds to the function presented in Eq. (4).

Note that, because of their intrinsically synthetic nature, the two rates *β* and *χ* just defined are very difficult to be quantified in real applications. Since both parameters are the product of several factors and cannot be derived by direct field observations, they have been estimated so as to obtain a good match between measured and simulated prevalence (see Section 4).

### 3.6 Hydrological connectivity

To model larval transport and snail dispersal, which represent the second layer of spatial connectivity, we define two row-stochastic matrices Z and T that describe the probability that a snail or a cercaria/miracidium moves between any two water points, respectively. The spread over the hydrological network is modeled as a biased random-walk process on an oriented graph (Bertuzzo, Azaele, Maritan, Gatto, Rodriguez-Iturbe, Rinaldo, 2008, Bertuzzo, Maritan, Gatto, Rodriguez-Iturbe, Rinaldo, 2007), where edges are represented by oriented segments according to the water flow direction between any two water points. Because of the slow water mixing within lakes, we assume that the water points along their perimeters are not spatially connected with each other (but they are connected to river in-/out-takes as appropriate).

The snail dispersal matrix $Z=[Z_{wi}]$ introduced in Eq. (3) is thus given by

(12) $$Z_{wi(w\neq i)}=\left\{ \begin{array}{cc} Z_{w}^{d} & \text{if}\;w\to i\text{,}\\ Z_{w}^{u} & \text{if}\;w\leftarrow i\text{,}\\ 0 & \text{if}\;w↮i\text{,} \end{array}\right.$$

where arrows indicate downstream connections, and $Z_{w}^{d}\left(Z_{w}^{u}\right)$ is the site-dependent fraction of snails moving along a downstream (upstream) edge. Given the set of neighbors connected to the generic water point *w*, the fractions $Z_{w}^{d,u}$ of snails moving outside the origin point are given by

(13) $$Z_{w}^{d,u}=\left\{ \begin{array}{cc} z^{d,u}/n_{w}^{d,u} & \text{if}\;n_{w}^{d,u}>0\text{,}\\ 0 & \text{if}\;n_{w}^{d,u}=0\text{,} \end{array}\right.$$

where *z^d^* [*z^u^*] is the probability that a snail leaving a point moves to another point along a downstream (outward edge) [upstream (inward edge)], and $n_{w}^{d}$ [$n_{w}^{u}$] is the outdegree [indegree] of water point *w*, i.e. the number of downstream [upstream] water points connected to *w*. Assuming that the transport process is conservative, i.e. $\sum_{i=1}^{n_{w}}Z_{wi}=1,$ in the inner nodes, where $n_{w}^{d}>0$ and $n_{w}^{u}>0,$ we can write that $n_{w}^{d}Z_{w}^{d}+n_{w}^{u}Z_{w}^{u}=1$. To close the specification of *Z_wi_*, we also need to define some specific conditions for headwaters and outlets of the hydrological network, and for water points along the lake perimeters. All the results presented in this work are obtained with reflecting boundary conditions, i.e. based on the probability *Z_ww_* to stay in one of such special points being defined as $Z_{ww}=1-\left(n_{w}^{d}Z_{w}^{d}+n_{w}^{u}Z_{w}^{u}\right),$ to preserve the conservativeness assumption. We note that, by definition, the reduced matrix that can be obtained by extracting the rows related to lake access points, where $n_{w}^{d}=0$ and $n_{w}^{u}=0$ (with exception for river confluence/divergence points), is an identity matrix. Snail dispersal is assumed to be independent of epidemiological status and origin site, hence $m_{w}^{S,E,I}=m$. According to other field studies, the mean distance moved by snails is 50 m/day (hence, over a distance of ≈ 2 km between any two water bodies, m=0.025/day), with a range of 30–110 m/day (Schneider, Frost, 1986, Schneider, Lyons, 1993). Results presented in Section 4 will consider the corresponding range of snail dispersal rates.

Larval transport matrix $T=[T_{wi}]$ is derived by using the same approach used for snails. The hydrological transport rates are assumed to be site-independent and equal for both free-swimming stages of parasites (cercariae and miracidia), hence $l_{w}^{C,M}=l$. Because of the limited locomotion of larval stages, transport is assumed to be unidirectional from upstream to downstream, with residence time in each water body of ≈  4.5 day (hence l=0.22/day). Similarly to snail dispersal rates, numerical results will be also shown considering different larval transport rates.

For the sake of simplicity, and to avoid introducing further hypotheses in our modelling scheme, we assume that the volume of water effectively accessible to snails and cercariae/miracidia is relatively homogeneous within the network, i.e. $V_{w}=\bar{V}\;\forall w$. In fact, snail hosts of schistosomes usually occur in shallow water near the shores of lakes, canals and rivers. Note that results are independent from the value assumed by $\bar{V},$ since water volumes appear in model [1] either as fractions *V_i_*/*V_w_* (Eqs. (3) and (5)) or together with the contamination rate *χ* (Eq. (4)), whose definition will also incorporate the volume effect.

### 3.7 Model outputs

As schistosomiasis is endemic in Senegal, model outputs are evaluated by simulating system [1] up to convergence to steady state starting from an initial hypothetical condition in which human communities are set to be equal to the community size in absence of the parasite ($N_{v}\left(0\right)=H_{v}$ in all villages), while all water points are infested by just one infectious snail ($S_{w}\left(0\right)=1/\gamma_{w}-1,$$E_{w}\left(0\right)=0,$ and $I_{w}\left(0\right)=1$ in all water points). Model [1] gives an estimate of the total number of adult parasites within each human community. We can thus define the mean worm burden *ω_v_* in each village (i.e. the average number of parasites per human host resident in *v*) as

(14) $$\omega_{v}=\frac{{\bar{P}}_{v}}{{\bar{N}}_{v}},$$

where a superscript bar indicates state variables at equilibrium. Note that the mean worm burden *ω_v_* includes also uninfected people. The estimation of a similar variable restricted to infected humans requires further steps and is computed as follows. First, we define the total number of human hosts resident in village *v* and carrying *p* parasites as

(15) $$h_{v}^{p}={\bar{N}}_{v}{\mathrm{NB}}_{v}^{p}\left(k,\omega_{v}\right),$$

where ${\mathrm{NB}}_{v}^{p}\left(k,\omega_{v}\right)$ is estimated according to a negative binomial distribution with clumping parameter *k* and mean *ω_v_*. As a second step, we introduce an infection threshold *τ* that represents the minimum parasite burden above which human hosts are considered to be infected. Specifically, we set *τ*=2, corresponding to one adult pair of parasites. Note that higher parasite loads may be required for schistosome reproduction to be effective (Gurarie et al., 2010). Therefore, in each village the mean worm burden of infected human hosts can be evaluated as the sum of the parasites carried by humans characterized by parasite burden larger than *τ*, divided by the total number of infected people, i.e.

(16) $$\omega_{v}^{\prime }=\frac{\sum_{p=\tau +1}^{{\bar{P}}_{v}}h_{v}^{p}p}{\sum_{p=\tau +1}^{{\bar{P}}_{v}}h_{v}^{p}},$$

where the denominator represents the total number of infected people in village *v*. We note that the mean worm burden is obviously found to be higher if estimated from infected humans only (i.e. $\omega_{v}^{\prime }>\omega_{v}$). From these definitions, it follows that the prevalence *u_v_* of infected human hosts in each village is given by

(17) $$u_{v}=\frac{\sum_{p=\tau +1}^{{\bar{P}}_{v}}h_{v}^{p}}{{\bar{N}}_{v}}.$$

We finally note that the mean worm burden and the infection prevalence can be easily upscaled to the overall study area via averaging, using village population sizes as weights. We term *ω_g_* ($\omega_{g}^{\prime }$) and *u_g_* the global mean worm burden and the global prevalence, respectively.

## 4 Results

### 4.1 Mean worm burden and prevalence distribution in Saint-Louis region

**Fig. 3:**
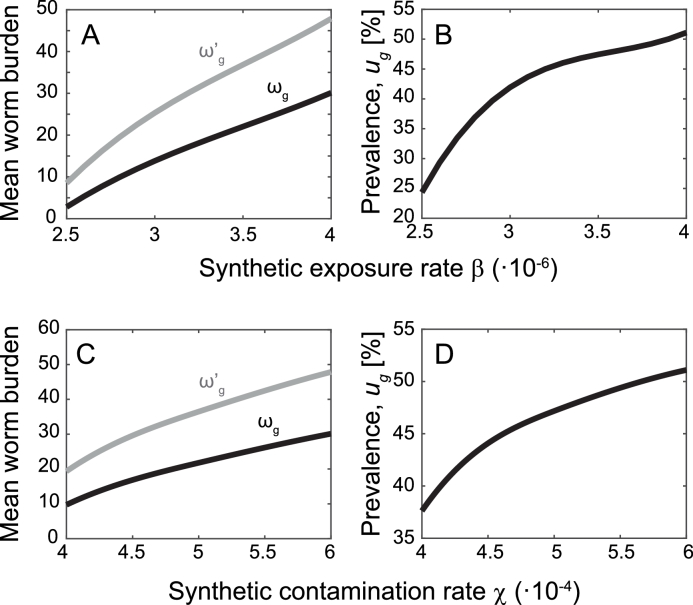
The effect of different infection and contamination levels on mean worm burden and prevalence within the whole pilot area of Fig. 2. (A) Simulated mean worm burden (averaged over the whole study area) estimated from all humans (*ω_g_*, black) and from infected humans only ($\omega_{g}^{\prime },$ grey) as a function of increasing values of the exposure rate *β* (parasites m^3^ cercariae${}^{-1}$ person${}^{-1}$ day${}^{-1}$). The contamination rate is set to $\chi =6\cdot 10^{-4}$ miracidia parasites${}^{-1}$ day${}^{-1}$. (B) Simulated human prevalence (averaged over the whole study area) as a function of *β*. C,D) As in A and B, for increasing values of the contamination rate *χ* (miracidia parasites${}^{-1}$ day${}^{-1}$). The infection rate is set to $\beta =4\cdot 10^{-6}$ parasites m^3^ cercariae${}^{-1}$ person${}^{-1}$ day${}^{-1}$. Simulations are obtained for snail dispersal rate m=0.025 day${}^{-1}$ with downstream dispersal probability $z^{d}=0.5,$ and larval transport rate l=0.22 day${}^{-1}$. All other parameters as defined in Section 3.

The prevalence and the mean worm burden in the pilot area obtained through model simulations is shown in Fig. 3. As mentioned in Section 3, the synthetic human exposure (*β*) and contamination (*χ*) rates are very difficult to be quantified in real applications. Clearly, increasing values of the two parameters are associated with increasing value of mean worm burden and human prevalence, because they directly act on the force of infection and the human contamination level, respectively.

**Fig. 4:**
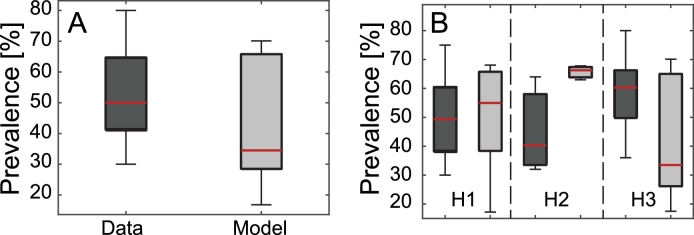
Prevalence comparisons between data and model simulations. (A) Boxplot of the prevalence estimated from data available in 24 villages (dark grey), and comparison with the output of the model in the same subset of villages (light grey). (B) Boxplots of the prevalence aggregated for the three habitats H1 (lakes), H2 (canals), and H3 (river). In each box, the central red mark is the median, the edges are the 25th and 75th percentiles, and the whiskers extend to the most extreme values. The outputs of the model are obtained by setting parameters as in Fig. 3. (For interpretation of the references to colour in this figure legend, the reader is referred to the web version of this article.)

Prevalence data within the study area are available only in 24 villages (see again Fig. 2, data available from the Upstream Alliance). Here, we refrain from a formal calibration of model [1]: rather, we explore ranges of *β* and *χ* that produce reasonable epidemiological outputs. Therefore, all numerical results are obtained by using exposure and contamination rates selected for better describing the qualitative prevalence distribution, in terms of mean and variance estimated from the subset of 24 sampled villages (Table 1). An ANOVA 1–way test performed on actual and simulated prevalences shows that differences in the mean are not significant (*p*–value >  0.05). A two-sample Kolmogorov-Smirnov test also confirms that differences in the distributions of data and model results are not significant (*p*–value >  0.05). Globally (i.e. averaging prevalence over the 24 sample villages), the model is able to qualitatively reproduce the mean prevalence distribution estimated from available data, yet it underestimates extreme values (Fig. 4A).

**Table 1:** Prevalence distribution statistics. Actual mean and variance are estimated from a subset of 24 sampled villages (see details in the text). The outputs of the model are obtained by setting $\beta =4\cdot 10^{-6}$ parasites m^3^ cercariae${}^{-1}$ person${}^{-1}$ day${}^{-1},$$\chi =6\cdot 10^{-4}$ miracidia parasites${}^{-1}$ day${}^{-1},$m=0.025 day${}^{-1},$$z^{d}=0.5,$l=0.22 day${}^{-1}$.

	Mean(*u_v_*)	Var(*u_v_*)
Data	0.53	0.022
Model	0.46	0.033

Averaged results over the three habitats H1 (villages close to lakes), H2 (villages close to canals), and H3 (villages close to main river courses), also show that the largest errors are obtained for villages close to canals (H2), where prevalence is systematically overestimated (Fig. 4B). Note, however, that only 3 villages of the sampled dataset are located in habitat H2. Another ANOVA 1–way test confirms that the differences in the mean of actual and simulated prevalences associated with the three habitats, limited to the 24 sampled villages, are not significant (*p*–value >  0.05).

### 4.2 Infection intensity

**Fig. 5:**
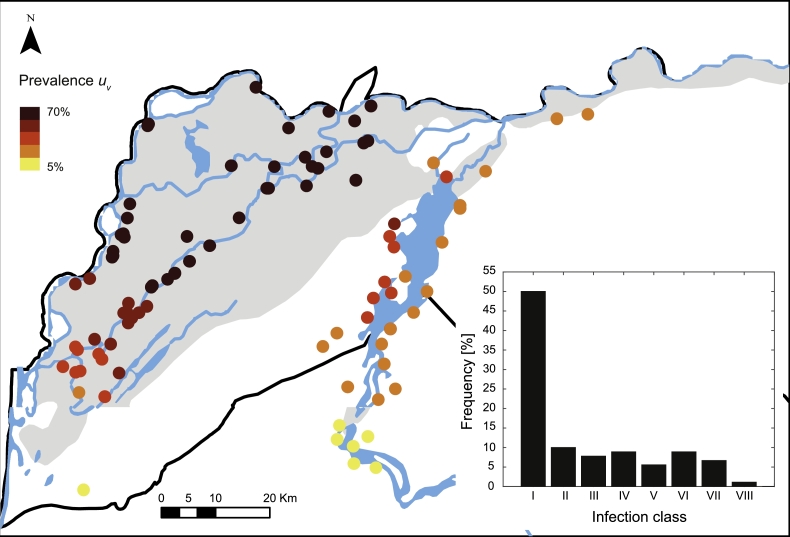
Spatial distribution of prevalence at the village level. In the inset, the frequency distribution of the infection classes. Classes I to VIII include villages with mean worm burden *ω_v_* up to 80 parasites per person. Simulations are obtained by setting parameters as in Fig. 3.

At the village level, the simulated spatial distribution of schistosomiasis prevalence in human hosts shows lower values in villages close to Lac de Guiers and in the southern part of the Senegal River system (Fig. 5). Although this spatial distribution at the village level cannot be supported by prevalence data (available only in a small subset of the villages under study), the frequency distribution of the mean worm burden as simulated by the model is consistent with field evidence available in the literature (see e.g. Gurarie et al., 2010). For the analysis, the mean worm burden can be usefully classified into different infection classes, based on intervals *Δp* of average parasite number within each human host (say, *Δp* = 10 parasites). Its frequency distribution shows a marked peak (≈ 50% of villages) in the lowest infection intensity class (class I, 0 ≤ *ω_v_* ≤ 10) (inset of Fig. 5), in accordance with parasites distribution usually found in literature (see e.g. Feng et al., 2004).

### 4.3 Hydrological regimes

**Fig. 6:**
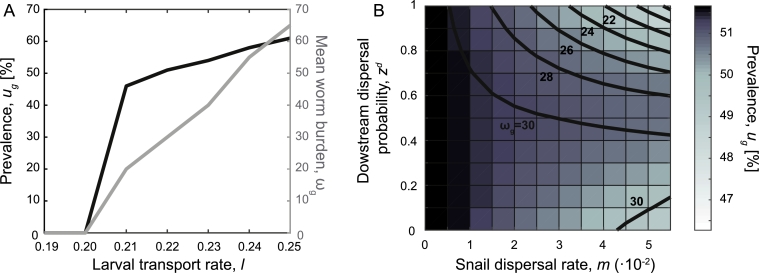
The effect of different hydrological regimes. (A) Simulated prevalence and mean worm burden (averaged over the whole study area) as a function of increasing values of the larval transport rate *l*. The snail dispersal rate is set to m=0.025 day${}^{-1},$ with downstream dispersal probability $z^{d}=0.5$.( B) Simulated prevalence *u_g_* obtained for different values of snail dispersal rate *m* and downstream dispersal probability *z^d^*. Black lines represent the contour lines of the mean worm burden *ω_g_*. The larval transport rate is set to l=0.22 day${}^{-1}$. All other parameters as in Fig. 3.

In absence of data on the typical hydrological regime of the study area, we investigate the effects of different conditions on the disease prevalence and the corresponding mean worm burden. Results show that, for the parameter settings used in model simulations, at low values of larval transport rate *l* the disease cannot persist in the population, whereas, for higher values, both the prevalence and the mean worm burden increase with *l* (Fig. 6A). Results also show that global infection is less strongly affected by snail dispersal, whose rate *m* is responsible for less than 5% of prevalence variation. The mean worm burden is found to increase with lower value of downstream dispersal probability *z^d^*, i.e. higher infection intensities are enhanced by snail upstream movements (Fig. 6B).

## 5 Discussion

In this work we have proposed a spatially explicit network model to describe schistosomiasis transmission dynamics in the endemic region of the Lower Basin of the Senegal River. Specifically, the model couples local-scale eco-epidemiological dynamics with spatial mechanisms related to human mobility, water-mediated snail dispersal and hydrological transport of schistosome larvae. Although a formal calibration of the model turned out to be impractical because of the high dimensionality of the problem and the still limited amount of information available for the pilot area, model parameters were set to be representative of the timescales, spatial distribution and intensity of infection involved in schistosomiasis transmission. It is thus noteworthy that model simulations produce epidemiological patterns that are consistent with field observations. Specifically, the model predicts higher schistosomiasis prevalence in the northern part of the pilot area, especially close to canals and main river courses. As expected, the mean worm burden is unevenly distributed among people, with most individuals hosting less than 10 parasites within their body.

In our framework, human mobility is assumed to drive potential water contact patterns which, in turn, determine human exposure to (and contamination of) environmental freshwaters. These patterns have been estimated by coupling the analysis of a large dataset of properly anonymized CDRs with hydrological information derived from satellite maps and geographic information systems. We note that actual water contact patterns can clearly be different from those estimated through big-data analytics, for a variety of reasons. First, local communities can be exposed to environmental sources of (possibly infested) freshwater other than those considered here (rivers, streams and lakes), i.e. small reservoirs, man-made irrigation canals and agricultural impoundments; second, the reasons why each village relies on a specific set of water points may go beyond sheer distance-based considerations, possibly involving local habits and traditions (in particular, preference towards contact with river water may explain the overestimation of prevalence in villages close to canals); third, the propensity and/or need to get in contact with environmental freshwater may be different for different socio-economic/age groups and possibly be subject to spatiotemporal variability (i.e. water-related behavior may change in specific seasons of the year, or be different for people who travel or who stay at home). All these sources of complexity have not been included in our analysis. However, we maintain that the approach explored in this paper can still offer a first-order approximation that can be useful in large-scale applications, as it can be made operative in a relatively straightforward way – provided that suitable data are available. We also note that the framework is general enough for the potential water contact matrix **Ω** to be able to accommodate actual data, namely if village-based surveys on water contact patterns are available.

Conversely, snail dispersal and larval transport represent physical mechanisms by which the pathogen can spread along waterways, thus providing an environmental route for the spatial propagation of the disease. Model simulations show that hydrologically mediated processes may have relevant impacts on the global prevalence and the relative mean worm burden, especially with respect to larval transport from upstream to downstream water points. The model has also shown the role of upstream snail dispersal, which is responsible for higher infection intensity. These findings call for a more in-depth description of the snail locomotory behavior and the peculiar hydrologic conditions of the region under study (OMVS, Organisation pour la mise en valeur du fleuve Sénégal, 2003). In particular, the locomotion of snails may be different according to their infection status (Boissier, Rivera, Moné, 2003, Alberto-Silva, Santos, Santos, Mello-Silva, 2015); also, the temporal variability of flood stages, as determined by seasonal rainfall, may have important implications for water contact patterns, as well for the life cycle of snail hosts and larval organisms. Even the environmental alterations induced by the construction of the Diama Dam need be addressed in the context of disease transmission (Southgate, 1997, Sow, De Vlas, Engels, Gryseels, 2002): on the one hand, in fact, the dam has changed the Senegal basin’s flood plain from a salty and brackish aquatic environment with marked seasonal changes to a low-flow perennial freshwater system; on the other, it has raised water levels in the upstream section of the river, thus creating reserves for irrigation (OMVS, Organisation pour la mise en valeur du fleuve Sénégal, 2003). Agricultural development, in turn, leads to increasing levels of chemical and biological pollution related to the discharge of wastewater and pesticides, with nontrivial effects for disease transmission dynamics (Rohr et al., 2008).

Although the limited amount of information available for the pilot area makes our analysis still preliminary, the modelling framework presented in this work is a promising tool for disease control and might be applied to other areas and water-related diseases. In fact, the current strategy for schistosomiasis control is mainly based on treatment with praziquantel, which is the only recommended drug for the infection caused by the schistosome infecting humans, but does not confer permanent immunity (Thétiot-Laurent et al., 2013). This makes the implementation of a comprehensive approach fundamental for fighting the disease. Such an approach must be based not only on mass chemotherapy, but also on human development, exposure and contamination prevention (e.g. by improving access to safe water, sanitation and hygiene; Ogden et al., 2014), awareness about risk factors (via information, education and communication campaigns, see e.g. Rollinson et al., 2013) and, possibly, biological control of snail intermediate hosts (Sokolow et al., 2015). In this respect, our model, properly informed by census, environmental, and malacological surveys, can help in identifying the focal hotspots of disease transmission. It can thus be a useful tool for evaluating the effectiveness of specific intervention strategies.
